# Early post-operative changes in quality of life and psychological symptoms after breast cancer surgery: the role of perceived pain, upper limb disability, and disease acceptance

**DOI:** 10.1007/s00520-026-11278-6

**Published:** 2026-09-30

**Authors:** Carlo Lai, Emanuele Giraldi, Teresa Paolucci, Alice Cichelli, Sofia Mazzani, Stefano Delli Pizzi, Giorgio Veneziani

**Affiliations:** 1 Department of Dynamic and Clinical Psychology, and Health Studies, Sapienza University of Rome, Via Degli Apuli, 1, 00185 Rome, Italy; 2 Department of Oral Medical Sciences and Biotechnology (DSMOB), “G. d’Annunzio” University of Chieti-Pescara, 66100 Chieti, Italy; 3 Center for Sport, Disability and Rehabilitation (CARES), “G. d’Annunzio” University of Chieti-Pescara, 66100 Chieti, Italy; 4 Biobehavioral Arts and Culture for Health, Sustainability and Social Cohesion (BACH), “G. d’Annunzio” University of Chieti-Pescara, 66100 Chieti, Italy; 5 Department of Theoretical and Applied Sciences (DiSTA), eCampus University, 22060 Novedrate, Italy; 6 Behavioral Imaging and Neural Dynamics Center (BIND), “G. d’Annunzio” University of Chieti-Pescara, 66100 Chieti, Italy; 7 Department of Neuroscience, Imaging, and Clinical Sciences, University «G. d’Annunzio» of Chieti-Pescara, 66100 Chieti, Italy; 8 Present Address: Departmental Simple Operating Unit of Breast Surgery, Interdisciplinary Group, “Santo Spirito” Hospital of Pescara, 65124 Pescara, Italy

**Keywords:** Breast cancer, Quality of life, Psychological symptoms, Upper limb disability, Pain, Disease acceptance

## Abstract

**Purpose:**

The present study aimed to evaluate changes in QoL and psychological symptoms (anxiety and depression) in women with BC between the first days (T0) and 1 month after surgery (T1). In addition, the study examined longitudinal associations of perceived pain, upper limb disability, and disease acceptance with QoL and psychological symptoms across T0 and T1.

**Methods:**

A prospective, observational cohort study was conducted on 101 women with BC. At T0 and T1, the EuroQol Five-Dimension Five-Level, the Hospital Anxiety and Depression Scale, the Numeric Rating Scale, the Quick Disability of the Arm, Shoulder and Hand, and an adapted version of the Chronic Pain Acceptance Questionnaire were administered. Data were analyzed using paired-samples *t*-tests and linear mixed models (LMMs).

**Results:**

The results showed increased QoL and decreased psychological symptoms between T0 and T1. The LMMs revealed that assessment time was not significantly associated with QoL and psychological symptoms once perceived pain, upper limb disability, and disease acceptance were considered. Notably, perceived pain and upper limb disability were negatively associated with QoL. In addition, upper limb disability was positively associated with depressive symptoms. Lastly, disease acceptance was negatively associated with both anxiety and depressive symptoms.

**Conclusion:**

Improvements in QoL and psychological symptoms may occur within the first month after BC surgery. At equivalent levels of perceived pain, upper limb disability, and disease acceptance, the timing of assessment seems to provide no additional information about QoL and psychological symptoms. The findings suggest the importance of timely, multidisciplinary rehabilitation care for women with BC during the early post-operative period.

**Supplementary Information:**

The online version contains supplementary material available at https://doi.org/10.1007/s00520-026-11278-6.

## Introduction

Breast cancer (BC) is one of the most common and debilitating malignancies and the leading cause of cancer-related mortality among women worldwide [1]. Advances in early diagnosis and surgical procedures have promoted substantial improvements in the prognosis and survival rates of women with BC [2]. In this context, breast-conserving surgery and mastectomy constitute the main surgical procedures for treating BC and reducing recurrence risk [3]. The post-operative period following surgery is characterized by multiple physical and psychological changes, representing a challenging experience with repercussions on the recovery of BC patients [4, 5]. Accordingly, the literature highlights the need to delve deeper into this period to identify specific indicators of therapeutic success and overall well-being, rather than focusing solely on survival [5–7].

Recent research has increasingly focused on investigating the quality of life (QoL) and psychological symptoms of women undergoing BC surgery in the post-operative period [6, 8]. Specifically, a recent systematic review and meta-analysis showed that QoL seems to follow a non-linear trajectory after BC surgery, ranging from transient declines to improvement and stabilization over time [6]. During the post-operative period, patients might recover from the acute effects of surgery and adapt to the implications of treatment, reducing the uncertainty and threat posed by the disease [6, 9]. Other studies highlighted that anxiety and depression symptoms tend to decrease in the early post-operative period after surgery [10–12], although the evidence is weaker and more heterogeneous compared to the long-term post-operative period [4, 7, 8, 13–15], thus requiring further investigation. In addition, a limited number of studies examined the influence of physical and psychological variables on the well-being of BC patients immediately after surgery [16–18]. Filling this gap appears necessary considering that physical recovery and psychosocial adjustment seem to play a key role in the trajectories of change of QoL and psychological symptoms following BC surgery [6, 15, 16].

In this regard, perceived pain, defined as the experience of pain severity reported by patients, and upper limb disability, which involves functional limitations and symptoms affecting the arm, shoulder, or hand, are among the most relevant variables in this context, commonly occurring in BC patients and leading to debilitating complications after surgery [19–22]. Indeed, both perceived pain and upper limb disability can contribute to difficulties in performing daily activities, decreased work capacity, and reduced social engagement, with a worsening of post-operative psychophysical outcomes [20, 22, 23]. Consistently, several studies showed that higher levels of perceived pain and upper limb disability were associated with decreased QoL and increased physical and psychological symptoms after BC treatments [21, 24–26].

In addition, it is important to consider that psychological processes can also play a significant role in influencing the post-operative recovery of BC patients [16, 18]. Specifically, disease acceptance, defined as the ability to acknowledge and experience cancer-related thoughts, emotions, and bodily sensations without excessive avoidance or struggle, seems to promote patients’ mental and physical well-being, as it allows them to engage in meaningful daily activities despite experiencing stressful and painful inner experiences related to the disease [27–30]. In this regard, previous studies showed that disease acceptance in BC patients was associated with higher levels of QoL after cancer treatment and lower levels of anxiety and depressive symptoms [28, 30–32]. The positive effects of disease acceptance would stem from greater psychological flexibility, which allowed patients to integrate disease-related impairments into their daily lives without controlling or avoiding them [27, 28].

Considering the research findings in this context, there is a need for further investigation not only of QoL and psychological symptom levels of women with BC during the early post-operative period, but also of how intra-individual physical and psychological factors may influence these outcomes. A deeper understanding of these issues could provide valuable insights for implementing timely, patient-centered intervention strategies soon after surgery, thus improving overall care for BC patients. Therefore, the present study aimed to evaluate changes in QoL and anxiety and depressive symptoms of women with BC during the early post-operative period, between the first days and one month after surgery. In addition, the study examined longitudinal associations of perceived pain, upper limb disability, and disease acceptance with QoL and anxiety and depressive symptoms across the early post-operative period.

## Materials and methods

### Participants and procedures

The participants of the present study were enrolled consecutively using a non-probabilistic sampling method from two inpatient Hospital Breast Units in Italy between February and July 2025. Specifically, the inclusion criteria were (1) age over 18 years; (2) women with a confirmed histopathological diagnosis of BC; (3) having undergone breast surgery (mastectomy or breast-conserving surgery) within a week; and (4) understanding of the Italian language. Exclusion criteria included (1) peripheral or central neurological disorders; (2) systemic diseases; (3) cognitive impairments; (4) recent myocardial infarction; and (5) previous cerebrovascular events.

The present study was approved by the Territorial Ethics Committee (protocol number: 53–2023) and was conducted in accordance with the Declaration of Helsinki (1964) and the Strengthening the Reporting of Observational Studies in Epidemiology (STROBE) statement guidelines [33] (Table S1, Supplementary Material). The medical staff of each hospital unit assessed patients’ eligibility according to the inclusion and exclusion criteria. All participants received detailed information about the study’s aims and procedures and provided their informed consent to participate. The study employed a prospective, observational cohort design with two longitudinal assessments. The first assessment (T0) was conducted within the first three days after breast surgery during hospitalization at the hospital unit. At T0, sociodemographic (age, marital status, education level, and occupational status) and clinical (smoking status, type of surgery, type of planned cancer treatment) data were collected, and self-report questionnaires were administered to assess QoL, anxiety and depressive symptoms, perceived pain, upper limb disability, and disease acceptance. The second assessment (T1) was conducted 1 month (30 days) after T0 during the routine follow-up visit at the hospital unit. At T1, the same self-report questionnaires used at T0 were administered. The T0 assessment was performed a mean of 2.41 days after surgery (range, 1–3 days), whereas the T1 assessment was performed a mean of 32.41 days after surgery (range, 31–33 days).

### Measures

The EuroQol Five-Dimension Five-Level (EQ-5D-5L) [34, 35] is a self-report questionnaire that assesses current overall health status and provides a measure of perceived health-related QoL. The EQ-5D-5L comprises the evaluation of five dimensions of health (mobility, self-care, usual activities, pain/discomfort, and anxiety/depression) and a visual analog scale (EQ-5D-5L-VAS). In the present study, to assess changes in patients’ global perceived QoL, the EQ-5D-5L-VAS was used as the primary measure of QoL. It asks respondents to rate their current health status on a vertical scale ranging from 0 (“worst health you can imagine”) to 100 (“best health you can imagine”). Higher scores on the EQ-5D-5L-VAS indicate higher perceived QoL. In addition, the EQ-5D-5L utility index scores were calculated from the five dimensions of the questionnaire using the Italian value set [36], with higher scores indicating higher health status.

The Hospital Anxiety and Depression Scale (HADS) [37] is a self-report questionnaire consisting of 14 items, rated on a 4-point Likert scale (0 = “not at all” to 3 = “most of the time”), that assesses anxiety and depressive symptoms. Specifically, the questionnaire consists of two subscales designed to assess anxiety (HADS-A) (7 items) and depression (HADS-D) (7 items). Higher scores on each subscale reflect higher levels of anxiety and depression symptoms, respectively. Scores ≥ 8 on each subscale were considered above the threshold for clinically relevant anxiety and depressive symptoms, respectively [38]. In the present study, the reliability coefficients (Cronbach’s *α*) of the HADS subscales were 0.85 (HADS-A) and 0.70 (HADS-D).

The Numeric Rating Scale (NRS) [39] is an 11-point self-report numeric scale ranging from 0 (“no pain”) to 10 (“worst possible pain”). The NRS evaluates the current pain intensity, with higher scores indicating greater perceived pain.

The Quick Disability of the Arm, Shoulder and Hand (QuickDASH) [40] is an 11-item self-report questionnaire with a 5-point Likert scale (1 = “no difficulty/symptom” to 5 = “extreme difficulty/inability to perform the activity/symptoms”). The questionnaire assesses the extent of disability and specific symptoms of the upper limbs, including the degree of difficulty in performing various physical activities due to problems with the shoulders, arms, and hands and the quality of sleep, social activities, daily activities, the intensity of pain, and numbness. Higher scores indicate greater disability in upper limb function. In the present study, the QuickDASH total score showed an excellent internal consistency (Cronbach’s *α* = 0.91).

The Chronic Pain Acceptance Questionnaire (CPAQ-8) [41] is a self-report questionnaire comprising eight questions, each rated on a 7-point Likert scale (0 = “never true” to 6 = “always true”). The questionnaire assesses the acceptance of chronic pain, including activity engagement, which refers to the perceived level of activity despite ongoing pain, and pain willingness, which reflects the willingness to tolerate pain without resorting to inefficient pain treatment strategies. In the present study, the CPAQ-8 was adapted to assess disease acceptance in BC patients, replacing the terms “pain” and “chronic pain” with the terms “cancer” (e.g., “Although things have changed, I am living a normal life despite my cancer”) or “disease” (e.g., “I lead a full life even though I have a disease”) and maintaining the original structure of the items, response format, and scoring system. The specific items of the adapted version of the CPAQ-8 are reported in Table S2 of the Supplementary Material. Higher scores indicate greater disease acceptance. The total score of the adapted version of the CPAQ-8 showed acceptable internal consistency (Cronbach’s *α* = 0.79).

### Statistical analysis

Initially, descriptive analyses were conducted on participants’ sociodemographic and clinical data. Additionally, descriptive analyses were performed on EQ-5D-5L utility index scores at T0 and T1.

To examine changes in QoL, anxiety, and depressive symptoms, paired-samples *t*-tests were performed, comparing scores at T0 and T1 for the EQ-5D-5L-VAS, HADS-A, and HADS-D. In addition, changes in perceived pain, upper limb disability, and disease acceptance were examined using paired-samples *t*-tests comparing NRS, QuickDASH, and adapted CPAQ-8 scores at T0 and T1. Effect sizes were assessed using Cohen’s *d*. A sensitivity power analysis was conducted using G*Power [42] to evaluate the smallest detectable effect size for paired-samples comparisons with the available sample of 101 participants. Assuming a two-sided paired-samples *t*-test, an *α* level of 0.05, and 80% power, the smallest detectable effect size was Cohen’s *d* = 0.28.

Subsequently, linear mixed models (LMMs) were conducted to assess longitudinal associations of perceived pain, upper limb disability, and disease acceptance with QoL and anxiety and depressive symptoms across T0 and T1. For each outcome assessed (EQ-5D-5L-VAS, HADS-A, HADS-D), an LMM was implemented, including assessment time (T0 vs. T1) and physical and psychological variables (NRS, QuickDASH, adapted CPAQ-8) as fixed effects, and age and type of surgery (breast-conserving surgery vs. mastectomy) as covariates. The analyses were controlled for age and type of surgery, as previous studies showed their effects on QoL and psychological symptoms in BC patients [6, 8, 9, 14]. A random intercept for participant ID was included to account for within-subject dependence. The LMMs were estimated using the Restricted Maximum Likelihood estimation method, and degrees of freedom were approximated using Satterthwaite’s method. Correlations among physical and psychological variables used in the LMMs were all < 0.70, indicating no multicollinearity violation (Table S3, Supplementary Material).

Statistical analyses were performed using JASP software [43]. A significance level of *p* < 0.05 was considered for all statistical analyses performed.

## Results

A total of 146 patients were assessed for eligibility. Of these, 12 declined to participate, and 33 were excluded before T0, as 22 exhibited medical or oncological complications that prevented their inclusion or completion of the study procedures, and 11 provided incomplete or non-evaluable questionnaires. The final cohort of participants enrolled in the present study comprised 101 women with BC who had undergone breast surgery. No missing data were identified for any of the variables examined, and no dropouts were observed between the T0 and T1 assessments. The sociodemographic and clinical characteristics of the participants are reported in Table 1.

**Table 1:** Sociodemographic and clinical characteristics of BC patients (*n* = 101)

Characteristics	*n* (%)
Age	
Mean (SD)	63.59 (11.76)
Range	36–87
Marital status	
Single	15 (14.9)
Married	70 (69.3)
Separated	4 (4.0)
Widowed	12 (11.9)
Educational level	
Elementary school	13 (12.9)
Middle school	24 (23.8)
High school	36 (35.6)
University	28 (27.7)
Occupational status	
Employed	50 (49.5)
Homemakers	28 (27.7)
Retired	23 (22.8)
Smoking status	
Smokers	6 (5.9)
Non-smokers	95 (94.1)
Type of surgery	
Mastectomy	17 (16.8)
Breast-conserving surgery	84 (83.2)
Type of planned cancer treatment	
Chemotherapy	32 (31.7)
Radiotherapy	55 (54.5)
Endocrine therapy	10 (9.9)
No cancer treatment	6 (5.9)

Type of planned cancer treatment categories were not mutually exclusive

Descriptive analyses for the EQ-5D-5L utility index yielded a mean score of 0.88 ± 0.18 (range, − 0.12–1.00) at T0 and 0.93 ± 0.17 (range, − 0.18–1.00) at T1. In addition, at T0, 12 participants (11.9%) reported scores above the clinical threshold on the HADS-A and 3 participants (3.0%) on the HADS-D. At T1, 9 participants (8.9%) reported scores above the clinical threshold on the HADS-A and 4 participants (4.0%) on the HADS-D.

**Fig. 1:**
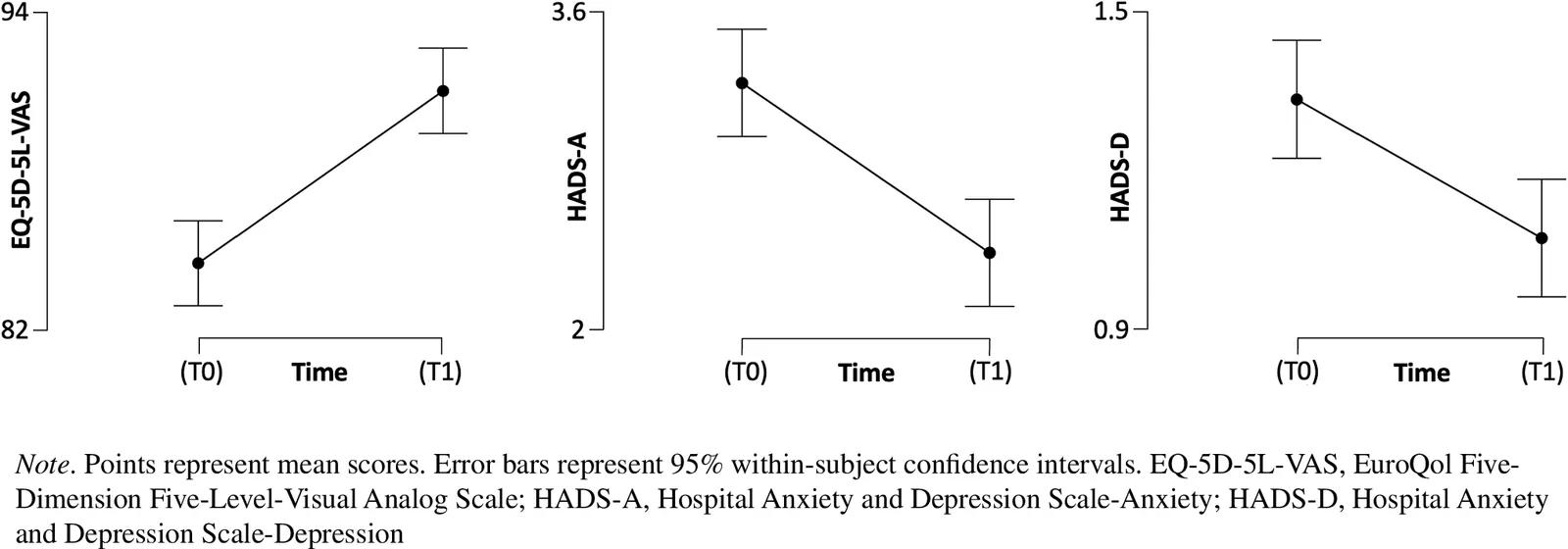
Descriptive plots of changes in QoL, anxiety, and depressive symptoms between the first days (T0) and 1 month after surgery (T1)

Paired-samples *t*-tests showed a significant increase in QoL over time from T0 (84.55 ± 11.47) to T1 (91.04 ± 7.76), with a mean increase of 6.49 points, *t*(100) =  − 5.69, *p* < 0.001, *d* = 0.57, 95% CI [0.36, 0.78]. In addition, anxiety symptoms decreased significantly from T0 (3.20 ± 3.65) to T1 (2.37 ± 3.44), with a mean decrease of 0.83 points, *t*(100) = 4.45, *p* < 0.001, *d* = 0.44, 95% CI [0.24, 0.65]. Lastly, depressive symptoms also decreased from T0 (1.32 ± 2.25) to T1 (1.06 ± 2.23), with a mean decrease of 0.26 points, *t*(100) = 3.30, *p* = 0.001, *d* = 0.33, 95% CI [0.13, 0.53]. The descriptive plots of paired-samples *t*-tests’ results are shown in Fig. 1.

Regarding changes in perceived pain, upper limb disability, and disease acceptance, paired-samples *t*-tests showed significant decreases in NRS scores from T0 (2.28 ± 1.83) to T1 (0.47 ± 1.24), *t*(100) = 10.25, *p* < 0.001, *d* = 1.02, 95% CI [0.78, 1.26] and in QuickDASH scores from T0 (14.35 ± 5.28) to T1 (13.76 ± 5.07), *t*(100) = 3.10, *p* = 0.003, *d* = 0.31, 95% CI [0.11, 0.51], as well as a significant increase in adapted CPAQ-8 scores from T0 (19.49 ± 3.15) to T1 (24.06 ± 3.44), *t*(100) =  − 9.63, *p* < 0.001, *d* = 0.96, 95% CI [0.72, 1.19].

The LMM conducted for QoL showed that perceived pain (*b* =  − 3.65, *p* < 0.001) and upper limb disability (*b* =  − 0.30, *p* = 0.012) were negatively associated with QoL across T0 and T1. In addition, age emerged as a significant covariate, showing a negative association with QoL (*b* =  − 0.14, *p* = 0.007). In contrast, assessment time was not significantly associated with QoL (*p* = 0.760), nor were disease acceptance (*p* = 0.900) or type of surgery (*p* = 0.844). Moreover, the results showed that disease acceptance was negatively associated with anxiety symptoms across T0 and T1 (*b* =  − 0.15, *p* < 0.001). Neither perceived pain (*p* = 0.937) nor upper limb disability (*p* = 0.757) showed significant associations with anxiety symptoms. Additionally, assessment time, age, and type of surgery were not significantly associated with anxiety symptoms (all *p* > 0.05). Lastly, upper limb disability was positively associated with depressive symptoms across T0 and T1 (*b* = 0.07, *p* = 0.025), while disease acceptance showed a negative association (*b* =  − 0.03, *p* = 0.044). Perceived pain did not show a significant association with depressive symptoms (*p* = 0.139), nor did assessment time, age, or type of surgery (all *p* > 0.05) (Table 2).

**Table 2:** Results of linear mixed models conducted with assessment time and physical and psychological variables (perceived pain, upper limb disability, disease acceptance) as fixed effects, and age and type of surgery as covariates, for each outcome assessed (QoL, anxiety symptoms, depressive symptoms)

Variables	Outcomes		
	EQ-5D-5L-VAS		
	Estimate (SE)	*t*	*p*
NRS	− 3.65 (0.36)	− 10.25	< 0.001
QuickDASH	− 0.30 (0.12)	− 2.55	0.012
CPAQ-8	0.02 (0.16)	0.13	0.900
Assessment time	− 0.39 (1.28)	− 0.31	0.760
Age	− 0.14 (0.05)	− 2.77	0.007
Type of surgery	− 0.30 (1.51)	− 0.20	0.844
	HADS-A		
NRS	0.01 (0.10)	0.08	0.937
QuickDASH	0.02 (0.06)	0.31	0.757
CPAQ-8	− 0.15 (0.04)	− 3.97	< 0.001
Assessment time	− 0.14 (0.29)	− 0.49	0.624
Age	0.04 (0.03)	1.37	0.175
Type of surgery	1.51 (0.90)	1.68	0.096
	HADS-D		
NRS	0.06 (0.04)	1.49	0.139
QuickDASH	0.07 (0.03)	2.26	0.025
CPAQ-8	− 0.03 (0.02)	− 2.04	0.044
Assessment time	0.05 (0.13)	0.38	0.706
Age	− 0.004 (0.02)	− 0.23	0.822
Type of surgery	0.94 (0.57)	1.66	0.101

*SE = *standard error, EQ-5D-5L-VAS = EuroQol Five-Dimension Five-Level-Visual Analog Scale, *HADS-A* = Hospital Anxiety and Depression Scale-Anxiety, *HADS-D* = Hospital Anxiety and Depression Scale-Depression, *NRS* = Numeric Rating Scale, *QuickDASH* = Quick Disabilities Arm, Shoulder and Hand. *CPAQ-8* = adapted version of the Chronic Pain Acceptance Questionnaire, Assessment time = T0 *vs*. T1, type of surgery = breast-conserving surgery vs. mastectomy; random intercept variance and residual variance for each model: EQ-5D-5L-VAS Var(intercept) = 8.24, EQ-5D-5L-VAS Var(residual) = 43.99, HADS-A Var(intercept) = 10.12, HADS-A Var(residual) = 1.61, HADS-D Var(intercept) = 4.26, HADS-D Var(residual) = 0.30

## Discussion

The present study evaluated changes in QoL and anxiety and depressive symptoms of women with BC during the early postoperative period, between the first days and one month after surgery. Moreover, the study examined longitudinal associations of perceived pain, upper limb disability, and disease acceptance with QoL and anxiety and depressive symptoms across the early postoperative period.

The results showed a significant increase in QoL and a decrease in anxiety and depressive symptoms 1 month after surgery. In addition, the linear mixed models revealed that assessment time was not significantly associated with QoL and anxiety and depressive symptoms, once perceived pain, upper limb disability, and disease acceptance were considered.

The present study supports the results of previous research, showing that QoL increases and anxiety and depressive symptoms decrease in the early post-operative period of BC patients [7, 12, 16]. After BC surgery, the threat posed by the disease may diminish, and the sense of control over one’s condition and hope for the future may increase, thus promoting greater psychophysical well-being [8, 9]. It is worth noting that participants already exhibited high QoL and low levels of anxiety and depressive symptoms at T0, suggesting relatively favorable adjustment from the first days after surgery. This early post-operative profile appears consistent with previous studies highlighting that levels of psychological symptoms may already be low or decrease rapidly immediately after BC surgery [10, 44]. In addition, regarding the clinical relevance of the observed changes one month after surgery, the improvement in QoL exceeded the 5-point minimal important difference reported in the literature for the EQ-5D-5L-VAS in cancer patients [45]. Differently, the reductions in anxiety and depressive symptoms were below the approximately 1.5-point minimal important difference reported in the literature for the HADS subscales [46]. The relatively limited changes in psychological symptoms should be considered in light of the already low HADS scores at T0, which may have restricted the magnitude of further improvement.

Notably, the study also highlighted that taking into account perceived pain, upper limb disability, and disease acceptance, the timing of assessment did not provide additional information about BC patients’ outcomes. This result should be interpreted considering the significant concomitant changes found for these variables one month after surgery, including decreases in perceived pain and upper limb disability and an increase in disease acceptance. In this regard, the non-significant association between timing of assessment and the outcomes was observed after accounting for physical and psychological variables that themselves changed over the same assessment interval. Within these models, perceived pain and upper limb disability were negatively associated with QoL, suggesting their relevant role for the well-being of BC patients after surgery. Consistently, previous research in this context showed that higher levels of pain and upper limb disability were associated with reduced QoL and overall well-being [21, 25, 26]. These results could be explained by the fact that patients’ ability to perform daily and social activities, as well as their physical function and mobility, may be negatively affected by pain and upper limb disability [20, 22, 23, 26]. Interestingly, the latter was also positively associated with depressive symptoms, suggesting that upper limb disability might be associated with feelings of helplessness and impaired autonomy, potentially undermining not only BC patients’ physical health but also psychological well-being [25, 26, 47].

The present study also shed light on the role of psychological adjustment to illness after BC surgery, showing that disease acceptance was negatively associated with both anxiety and depressive symptoms. This finding supports and extends previous studies’ results [30–32], suggesting that the possibility of accepting the disease condition could be associated with BC patients’ psychological well-being since the early post-operative period. In this regard, integrating the disease into one’s life experience could promote a decrease in psychological symptoms soon after surgery by limiting rigid control strategies or avoidance behaviors and potentially reducing cancer-related anxious concerns and catastrophizing [27–30].

Lastly, it is worth noting that age was negatively associated with QoL. This result would seem to suggest that older women with BC may report a less favorable perception of their health during the early post-operative period. It could be hypothesized that older patients report lower QoL, potentially due to slower functional recovery and greater comorbidity compared to younger patients, as highlighted by a recent meta-analysis [6]. However, further studies that include and compare a larger number of clinical data are needed to support this result.

The study’s findings might suggest several clinical implications for women with BC undergoing surgery. Given the relevance of both physical and psychological variables identified in the present study, it may be important, from a clinical perspective, to take these factors into account in the care pathway for BC patients during the early post-operative period. Routine psychophysical assessments could be integrated into immediate post-operative care to identify potential patients at risk of difficulty in adapting to their condition and tailor interventions accordingly [8]. In addition, structured rehabilitation programs could be considered, with progression appropriate to surgical healing and individual functional status. Personalized physiotherapy approaches focused on active exercise and multimodal rehabilitation might also improve post-operative pain perception and upper limb mobility [48–50], factors that were associated with QoL and depressive symptoms in the present study. Furthermore, considering the negative associations found between disease acceptance and anxiety and depressive symptoms, it could be relevant to implement psychological interventions focused on the ability to integrate the experience of illness into one’s life [51, 52]. In this regard, interventions based on Acceptance and Commitment Therapy may be potentially suitable during the early post-operative period, as they aim to increase psychological flexibility through processes such as acceptance of difficult internal experiences, contact with the present moment, the self as context, clarification of values, and committed action [27, 52]. Overall, the present study suggests the importance of integrated collaboration between physiatrists and psychologists to understand and promptly address the interplay between physical and psychological factors in the early post-operative rehabilitation of women with BC.

Despite the relevance of the present findings, several limitations should be acknowledged. Firstly, the study enrolled participants using a consecutive, non-probabilistic sampling strategy from only two breast units. Furthermore, most participants were married, and radiotherapy was the most frequent type of planned cancer treatment. Therefore, the study’s results may not be generalizable to the broader population of women with BC or to other clinical settings. Secondly, all variables were assessed using self-report questionnaires, which are susceptible to multiple biases, including social desirability and response style biases. In addition, disease acceptance was assessed using an adapted version of the CPAQ-8, a measure originally developed for chronic pain acceptance assessment, potentially introducing construct validity bias. Thirdly, the study’s observational design does not allow causal inferences about the observed longitudinal associations between physical and psychological variables and outcomes. Furthermore, physical and psychological variables and outcomes were assessed simultaneously at the same assessment time points, preventing conclusions about the temporal direction of the associations. In addition, the study did not include a pre-operative assessment and was limited to two assessment time points over a relatively short follow-up period. Therefore, the assessment time points used prevent conclusions about changes from the patients’ pre-surgical status and limit the characterization of long-term trajectories of change in the outcomes. Lastly, although the study controlled the analyses for certain covariates (i.e., age and type of surgery), several clinical variables that could have influenced the results were not assessed (e.g., surgical success and complications, and analgesic regimens). Future studies should use probabilistic sampling strategies and recruit participants from multiple centers to achieve larger, more heterogeneous samples. Moreover, hetero-administered measurement tools and psychometrically validated questionnaires to assess disease acceptance would be needed. Further research should also include pre-operative assessments, additional assessment time points, and longer follow-up periods to more accurately examine changes in outcomes during the early post-operative period and to compare them with longer-term changes. Finally, it would be important to consider the influence of further clinical variables on the outcomes examined to support the results of the present study.

## Conclusion

In conclusion, the present study showed improvements in QoL and in anxiety and depressive symptoms of women with BC within the first month after surgery. In addition, the results highlighted that, once perceived pain, upper limb disability, and disease acceptance are taken into account, the timing of assessment appears to provide no additional information about QoL and psychological symptoms during the early post-operative period. In this regard, perceived pain and upper limb disability showed negative associations with QoL. In addition, upper limb disability showed a positive association with depressive symptoms. Lastly, disease acceptance showed negative associations with both anxiety and depressive symptoms. Overall, these findings suggest the relevance of timely and multidisciplinary rehabilitation care for women with BC who have undergone surgery, addressing both physical and psychological factors in the early post-operative period.

## Supplementary Information

Below is the link to the electronic supplementary material.

ESM 1 (DOCX 32.8 KB)

## Data Availability

The data are available upon reasonable request from the corresponding author.
